# Ovarian ependymoma presenting in pregnancy: a case report and literature review

**DOI:** 10.1186/s12884-020-03408-7

**Published:** 2020-11-18

**Authors:** Bo Jin, Jingjing Jiang, Hongfa Peng

**Affiliations:** 1 Department of Obstetrics and Gynecology, Second hospital of Hebei medical university, Heping West Road No. 215, Hebei province 050000 Shijiazhuang City, China; 2 Department of Obstetrics and Gynecology, Hebei General Hospital, 050051 Shijiazhuang, China

**Keywords:** Ovarian ependymoma, Oestrogen, Pregnancy, Hormone-based therapies, Case report

## Abstract

**Background:**

Ovarian ependymoma is a rare malignancy. Because of the extreme rarity, certain features of the neoplasm, including its clinical behaviour and optimal therapy, are incompletely characterized.

**Case presentation:**

A 32-year-old pregnant woman at term presented with a left ovarian neoplasm that occurred in the early stage of pregnancy. She underwent left adnexectomy during the caesarean section, and the neoplasm was histologically and immunohistochemically identified to be ovarian ependymoma. Immunohistochemical staining with oestrogen receptors and progesterone receptors showed strong positive staining. According to reports in the literature, the pathological type of ovarian ependymoma in our patient was the extra-axial type. Interestingly, the foetus was also found to have bilateral ependymal cysts during pregnancy. The patient received no further surgical treatment or adjuvant therapy. She and her 14-month-old baby both have no evidence of disease at present. The follow-up of both mother and child is still continuing.

**Conclusions:**

The case presented here illustrates high levels of oestrogen during pregnancy may stimulate viable malignant ependymal cells to proliferate. Hence, young women with extra-axial-type ependymomas may not be suitable for fertility preservation. Moreover, hormone-based therapies can be a potentially effective treatment for women with extra-axial ependymomas.

## Background

Ovarian ependymoma is an extremely rare gynaecologic malignancy that poses numerous diagnostic and treatment challenges. Because of the extreme rarity, certain features of the neoplasm, including the clinical behaviour, molecular profile, and optimal therapy, are incompletely characterized. Herein, we present a case of primary ependymoma of the ovary that appeared after pregnancy and grew gradually during pregnancy as well as a summary of primary ovarian ependymomas based on a review of all such cases published in the English literature.

## Case presentation

**Fig. 1:**
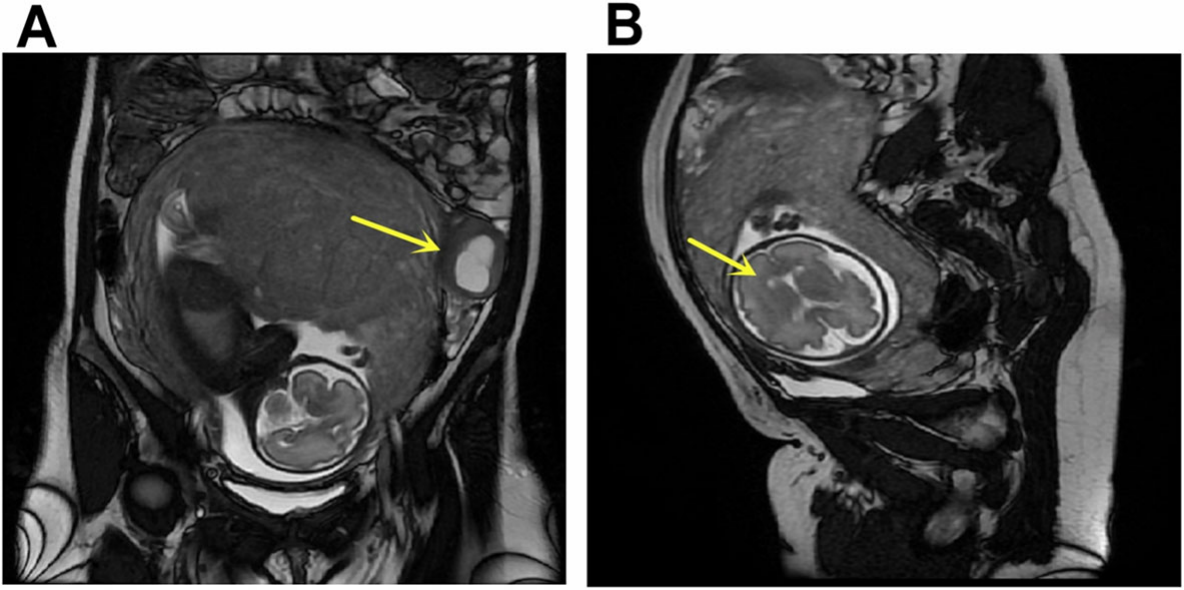
MRI shows a large mass on left ovary(**a**) and bilateral subependymal cysts(**b**)

**Fig. 2:**
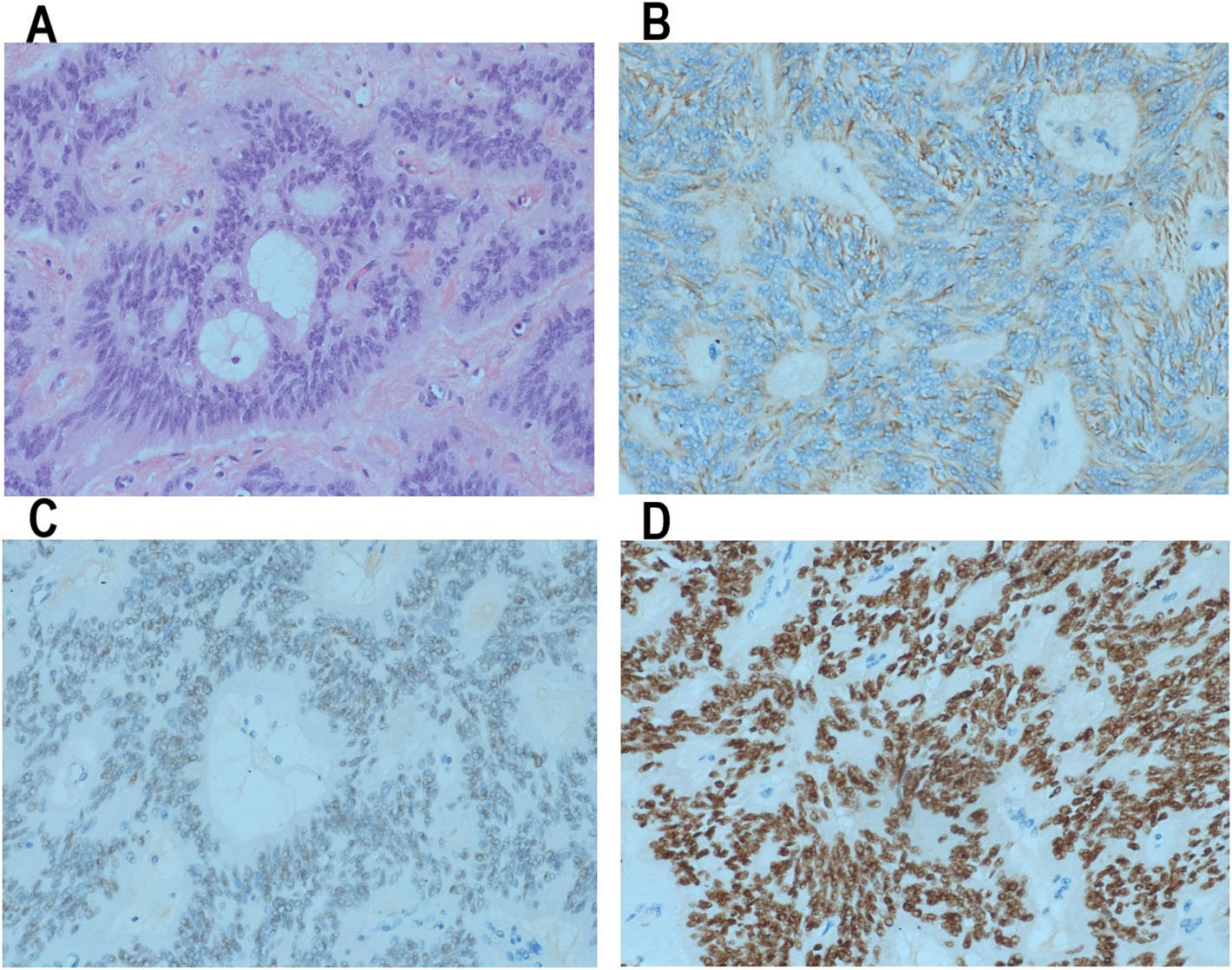
Anaplastic ependymoma showing multiple architectural patterns, including classical areas with perivascularpseudorosettes(**a**). Tumor cells were postitive for GFAP(**b**), ER(**c**), PR(**d**)

A 32-year-old woman (gravid 2,para 1) at term presented at our institution with a mass on her left ovary and previous caesarean section requiring a caesarean section. Her general medical and gynaecologic histories were unremarkable. In 2015, she had a caesarean section because of a foetal diaphragmatic hernia. Prior to this pregnancy, both gynaecological examination and transvaginal ultrasonography were negative. Transvaginal ultrasonography revealed an anechoic cyst in the left adnexal region at 45 days pregnant, with a size of approximately 2.6*2.4 cm. Subsequently, several pelvic ultrasound examinations were performed during the pregnancy, revealing that the cyst was enlarged and gradually became a cystic solid mass; colour doppler flow imaging suggested the color flow absence in the mass. In addition, ultrasound examination also showed that there was no echo area at the anterior corner of the foetal side of the ventricle at 28 weeks and 5 days of gestation. Then, foetal magnetic resonance imaging(MRI) was performed for further evaluation. A cystic solid soft tissue mass was demonstrated in the left adnexal region measuring 6.14*5.83 cm(Fig. 1a). Hypointense septation was also noted within the mass. MRI also suggested the presence of bilateral subependymal cysts in the foetus, and the larger cyst was approximately 0.4*0.3 cm in size(Fig. 1b). The serum CA125 level was 44.95 U/ml, and the AFP level was 248.0 ng/ml. At 37 weeks and 1 days of gestation a caesarean section was performed first. Then, surgical exploration showed that there were no ascites, and the enlarged left ovary was a solid cystic mass with a size of approximately 7 cm*5 cm*5 cm. The ovarian cyst was intact, had a smooth surface, and did not adhere to the surrounding tissues. Left salpingo-oophorectomy was performed. Microscopic examination indicated mature brain tissue in addition to neural tissues with perivascular pseudorosettes (Fig. 2a). Immunohistochemical staining with glial fibrillary acidic protein(GFAP) showed strong positive staining in the cytoplasm (Fig. 2b). The neoplastic cells were also positive for oestrogen receptors (Fig. 2c), progesterone receptors (Fig. 2d), vimentin, Ki-67, WT-1, CD10 and β-catenin; the cells were negative for CK7, CKpan, EMA, CD34 and α-fetoprotein. The patient was finally diagnosed with ovarian ependymoma. She had no fertility requirement, and we recommended further staging surgery. The patient refused our surgical recommendations and asked for follow-up. The literature has reported that aromatase inhibitors are effective in the treatment of patients with ovarian ependymoma. We advised her to take aromatase inhibitors and follow-up. She refused to take aromatase inhibitors because breastfeeding was not allowed while taking aromatase inhibitors. She and her 14-month-old baby both have no evidence of disease at present.

## Discussion and conclusions

Ependymoma is a type of glioma with differentiation of ependymal cells that usually arises in the central nervous system. The occurrence of ependymoma in the ovary is extremely rare. Kleinman et al. reported the first case of ovarian ependymoma in 1984 [1]. Only 34 cases have been reported since then. To the best of we know, this is the second reported case of ovarian ependymoma presenting in pregnancy. Owing to the rarity and multiple histologic patterns of ovarian ependymoma, diagnosis is challenging. Ependymoma of the ovary is mainly diagnosed via histology. The histopathologic resemblance of ovarian ependymoma to that of many primary or metastatic ovarian neoplasms makes diagnosis difficult. The key diagnostic feature of ovarian ependymomas is a microscopic perivascular pseudorosette and strong tumoural immunostaining for GFAP [2, 3].The treatment of ovarian ependymoma is also challenging due to the rarity of this tumour. The WHO Classification of Tumours of Female Reproductive Organs categorizes ovarian ependymomas as “neuroectodermal tumours” under the classification of “monodermal teratomas and somatic-type tumours arising from dermoid cysts” [4]. Hence, in the literature, the treatment of ovarian ependymoma is primarily based on the treatment of malignant germ cell tumours, including surgical debulking followed by adjuvant chemotherapy. The traditional first-line chemotherapy regimen consisting of bleomycin, etoposide and cisplatin(BEP) for germ cell tumours is effective for ovarian ependymomas [1, 5]. Moreover, Hino et al. have suggested that paciltaxel, ifosfamide, and cisplatin therapy can be used as an effective second-line therapy for ovarian ependymomas resistant to BEP therapy [5].

Early studies report that almost all patients with ovarian ependymomas received standard tumour surgery and adjuvant chemotherapy and had a favour-able prognosis even at an advanced stage. Carlsson et al. reported a case of ovarian ependymoma, where a patient who had experienced multiple instances of recurrence and multiple treatments had survived for 51 years when their case was reported [6]. To date, only two patients reported in the literature died of ovarian ependymoma [1, 7]. Ovarian ependymomas are reported among women mainly reproductive age groups. When a good prognosis has been achieved, the controversial and challenging problem arises of whether fertility can be retained in young women with ovarian ependymoma. In 2005, Takano et al. reported the first case of a woman with ovarian ependymoma who underwent fertility-sparing surgery and received adjuvant chemotherapy, and she achieved disease-free survival of 16 months [8]. Since then, a total of 3 patients with ovarian ependymoma have been reported to have received fertility-sparing surgery. All four patients received adjuvant chemotherapy. Unfortunately, three of the four patients experienced tumour recurrence in a short time. The mean time of tumour recurrence was 7.3 months(range 2–12 months). No pregnancy or fertility data were recorded in the four reports. Additional details are included in Table 1. The four patients all had advanced tumours with positivity for ERs and PRs. This means that all four patients had “extra-axial-type” ependymomas, which display aggressive behaviour [2]. Stolnicu et al. reported two types of ovarian ependymoma: central and extra-axial types [2]. The central type ependymoma with a central nervous system phenotype is thought to originate from the nervous tissue of teratomas and behave in a benign fashion. The extra-axial type ependymoma with a predominant microcystic and anaplastic pattern is thought to originate from pluripotent stem cells present in müllerian tissues and behave in an aggressive fashion [9]. Immunohistochemistry shows a marked difference between central and extra-axial ependymoma, the latter only being positive for ERs, PRs, epithelial membrane antigen, cytokeratin 34βE12, cell adhesion molecule 5.2, and cytokeratin 7. Some previous reports have shown that extra-axial ependymomas have widespread abdominal metastasis and frequently recur in a short time after complete surgery [2, 10–12]. According to reports in the literature, the pathological type of ovarian ependymoma in our patient was the extra-axial type. Since the ependymoma was diagnosed during the postpartum period, she refused staging surgery and the follow-up is still continuing.

**Table 1:** Summary of reported cases of ovarian ependymomas following fertility preservation surgery

Author/Year	Age	FIGO Stage	Treatment		ER and ER	CA125	Recurrence time	Outcome
			**Initial treatment**	**Adjuvant Therepy**				
Takano/2005 [8]	**23**	**IIIC**	**LSO,CT,FPCS**	**BEP**	**positive**	**640 U/ml**		**NED,16mo**
Stolnicu S/2011	**32**	**IIIB**	**LSO + PLNB,CT**	**PE**	**positive**	**94 U/ml**	**8mo**	**NED,36mo**
Simona /2011	**22**	**IIIA**	**FPCS,CT**	**CE**	**positive**		**12mo**	**AWD,30mo**
Hion M/2016	**21**	**IIIC**	**FPCS,CT**	**BEP,TIP**	**positive**	**209 U/ml**	**2mo**	**NED,24mo**

*FPCS F*ertility-preserving cytoreductive surgery, *CT *Chemotherapy, *PLNB *Pelvic lymph node biopsy, *RT *Radiotherapy, *AWD *Indicates alive with disease, *BEP *Bleomycin, etoposide, cisplatin, *PE *Etoposide, cisplatin, *CE *Carboplatin etoposide, *NED *No evidence of disease

In the literature, there is one hypothesis for the pathogenesis of extra-neural ependymomas in which misdirected primordial germ cells form ependymal tumours under the influence of female hormones [13]. In 1988, Auerbach et al. found that ovarian ependymoma tissues were rich in oestrogen and progestin, and they thought hormones may play a role in the treatment of ovarian ependymoma [14]. Subsequently, some studies also suggested that oestrogen and progesterone may promote the development of ovarian ependymoma [12, 14, 15]. For example, bilateral ER-positive ovarian ependymomas were reported in a pregnant woman by Carr et al. They suggested that the female hormonal responsiveness of ovarian ependymoma may be pathogenically significant [12]. In Auerbach et al’s report, one patient with ovarian ependymoma experienced recurrence while she was receiving hormone replacement therapy after oophorectomy, and the recurrent tumour was positive for both ERs and PRs. This suggests that hormone replacement therapy may stimulate viable malignant ependymal cells to proliferate [14]. Instead, oestrogen synthesis inhibitors have been shown to be effective against ependymoma. For example, Deval et al. showed that aromatase inhibitor treatment could be effective in cases of extra-axial ependymoma with prominent oestrogen receptor expression [10]. Gorski et al. reported a patient with advanced ovarian ependymoma who underwent sub-optimal surgical debulking followed by adjuvant chemotherapy with BEP, which resulted in a partial response. Due to extensive residual tumour tissues, the patient was maintained on anastrozole for over fifteen months without increased tumour burden [16]. Hence, high levels of oestrogen during pregnancy may stimulate viable malignant ependymal cells to proliferate. Young women with extra-axial-type ependymomas may not be suitable for fertility preservation.

In addtion, a case of ovarian ependymoma responding to gonadotropin-releasing hormone(GnRH) therapy was reported by Fang et al. in 2015, in which a 30-yr-old female with a large pelvic ependymoma who was treated with presurgical GnRH analogue therapy and fertility-sparing surgical resection remained disease-free after over 30 months without post-surgical treatment [17]. Similarly, some studies have demonstrated the role of anti-oestrogens in the treatment of ovarian ependymoma. For example, Yoffe et al. reported a case of recurrent ependymoma responding to tamoxifen [9]. These studies show that conservative, hormone-based therapies may be considered as a treatment option for patients with ovarian ependymoma.

The case illustrates high levels of oestrogen during pregnancy may stimulate viable malignant ependymal cells to proliferate. Young women with extra-axial-type ependymomas may not be suitable for fertility preservation. Hormone-based therapies can be considered as a treatment option for patients with extra-axial ependymomas.

## Data Availability

The datasets used during the current study are available from the corresponding author on reasonable request.

## Abbreviations

MRI: Magnetic resonance imaging
GFAP: Glial fibrillary acidic protein
BEP: Bleomycin, etoposide and cisplatin
ER: Estrogen receptor
PR: Progesterone receptor
GnRH: Gonadotropin-releasing hormone
